# Color Representation Is Retinotopically Biased but Locally Intermingled in Mouse V1

**DOI:** 10.3389/fncir.2017.00022

**Published:** 2017-03-29

**Authors:** Shuhei Aihara, Takashi Yoshida, Takayuki Hashimoto, Kenichi Ohki

**Affiliations:** ^1^Department of Molecular Physiology, Graduate School of Medical Sciences, Kyushu UniversityFukuoka, Japan; ^2^Department of Physiology, The University of Tokyo School of MedicineTokyo, Japan; ^3^Core Research for Evolutionary Science and Technology (CREST), Japan Science and Technology AgencyTokyo, Japan

**Keywords:** color, mouse, visual cortex, functional architecture, two-photon imaging, wide field Ca^2+^ imaging

## Abstract

Dichromatic vision is common in many mammals. However, color processing in the primary visual cortex (V1) of dichromatic mammals is relatively unknown compared to the trichromatic primates. In this study, we investigated the functional organization of color processing in mouse V1. The mouse retina has a graded expression pattern of two opsins along its dorsoventral axis. However, it is not clear whether and how this expression pattern is reflected in the cortical representation at local (several hundred microns) and areal (V1) level. Using *in vivo* two-photon calcium (Ca^2+^) imaging and wide-field Ca^2+^ imaging, we revealed that V1 neurons responded to S (UV)- and M (green)-opsin isolating stimuli with slightly biased color preference depending on retinotopic position in V1. This was consistent with the distribution of retinal opsins. At the cellular level, preferences for S- and M-opsin isolating stimuli were intermingled in a local region encompassing several hundred microns. These results suggest that functional organizations of color information are locally intermingled, but slightly biased depending on the retinotopic position in mouse V1.

## Introduction

Color provides important information for our lives. In many mammalian species, except for trichromatic primates (De Valois, 1960; Wiesel and Hubel, 1966; Livingstone and Hubel, 1984; Hendry and Reid, 2000; Chatterjee and Callaway, 2003), dichromatic color vision is common (Loop et al., 1987; Neitz and Jacobs, 1989; Jacobs et al., 1991; Calderone and Jacobs, 2003). It is relatively unknown, however, how color is processed in the cerebral cortex of dichromatic mammals (Hammond, 1971; Heimel et al., 2005; Ekesten and Gouras, 2008; Johnson et al., 2010; Tan et al., 2015). The basic visual functions of mouse are comparable to other mammals (Huberman and Niell, 2011) including dichromatic color vision. Therefore, studying color processing in mouse helps us to understand the basis of dichromatic color processing.

The mouse uses color information for behavior (Jacobs et al., 2004), and its dichromatic vision depends on short (S) and middle (M)-wavelength opsins with peak spectral sensitivities of 370 nm (UV) and 510 nm (green), respectively (Jacobs et al., 1991). The majority of cones coexpress these two opsins. Moreover, like many other animals, the expression of opsins is not uniform across the mouse retina (Temple, 2011). S opsins are dominantly expressed by cones in the ventral retina, whereas M opsins are dominantly expressed by cones in the dorsal retina (Szél et al., 1992; Applebury et al., 2000). Cones express both opsins in a graded manner along the dorsoventral axis. Higher expression of M opsins in the dorsal region and S opsins in the ventral region gradually decrease towards ventral and dorsal region, respectively, although S opsin is expressed at a relatively constant level (Röhlich et al., 1994; Lyubarsky et al., 1999; Applebury et al., 2000). Consistent with the expression pattern of opsins, retinal ganglion cells exhibit biased color responses depending on their retinal locations (Ekesten et al., 2000; Ekesten and Gouras, 2005; Wang et al., 2011).

Information of retinal position is preserved retinotopically in the V1. Therefore, the expression pattern of opsins in the retina may be reflected in the areal color representation of V1. However, it is controversial whether the gradient of opsin expression in the retina is reflected in the color representation in V1. By recording responses to color stimuli through V1, an electrophysiological study found that the distribution bias of UV- and green-light responsive neurons in V1 tend to be consistent with the bias in the retina (Ekesten and Gouras, 2008). On the other hand, a two-photon imaging study has reported no bias of UV and visible light responsive neurons by recording from lateral and medial V1 (~3 mm lateral, 0.5 mm anterior to lambda and ~2 mm lateral, 0.5 mm anterior to lambda; Tan et al., 2015). Besides these inconsistent results, the single unit electrophysiology in the former study is difficult to completely cover the entire V1, whereas recording sites in the latter study is relatively limited to small parts of V1. Thus, a complete map of color representation of mouse V1 remains to be elucidated. Furthermore, although the intermingled “salt and pepper-like” spatial organization of UV- and visible light-responsive neurons has been reported (Tan et al., 2015), it is unknown how the “salt and pepper-like” functional organization of color processing is affected by the bias of areal color representation, if color representation in the V1 is biased depending on the retinal opsin’s gradient.

Previous studies (Ekesten and Gouras, 2008; Tan et al., 2015) used UV and green light to test color selectivity, but stimulation with UV light activates both S and M opsins because M opsin has weak side-band sensitivity to UV light in addition to its peak at green wavelengths (Sun et al., 1997; Yokoyama et al., 1998; Lyubarsky et al., 1999; Govardovskii et al., 2000; Nikonov et al., 2006). To understand how color information is processed according to local and areal organization in the mouse cortex, we examined the neural responses to S- and M-opsin isolating stimuli (Estévez and Spekreijse, 1982) in V1 using two-photon Ca^2+^ imaging and wide-field Ca^2+^ imaging.

In this study, we found that many neurons responded to two opsin-isolating stimuli with diverse preference. These responsive neurons were not evenly distributed within V1 region but exhibited a biased distribution along the anteroposterior axis, consistent with the distribution of opsins in the retina; with more neurons that strongly responded to S-opsin isolating stimulation in the posterior V1, which retinotpically corresponds to the ventral retina where S opsins are highly expressed. Consistent with this, wide-field imaging revealed a color preference bias through entire V1. In contrast, neurons with diverse color preference were spatially intermingled in the range of several hundred microns. Thus, our results reveal the functional organization of color information in the mouse V1 at a cellular level and areal scale.

Preliminary results of this work were published in an abstract form (Aihara et al., 2013).

## Materials and Methods

All experiments were conducted in accordance with the institutional animal welfare guidelines of the Animal Care and Use Committee of Kyushu University and approved by the Ethical Committee of Kyushu University.

### Two-Photon Imaging

C57BL/6 wild type mice (postnatal days 60–90, Japan SLC, Hamamatsu, Shizuoka, Japan) were prepared for two-photon imaging as described previously (Ohki et al., 2005; Murakami et al., 2015). Mice were anesthetized with isoflurane (3.0% for induction, 1.5%–2.0% during surgery, and 0.5%–1.0% during imaging). To prevent dehydration, the eyes were coated with a thin layer of silicone oil. After incision of the skin, a metal plate for head fixation was attached to the skull using a dental adhesive (Super-bond, Sun Medical, Moriyama, Shiga, Japan). A small craniotomy was made over the visual cortex, and the underlying cortex was covered with artificial cerebrospinal fluid (ACSF) (150 mM NaCl, 2.5 mM KCl, and 10 mM HEPES (pH 7.4)). Mice were kept with their eyes open during imaging. A total of 0.8 mM Oregon Green 488 BAPTA-1 AM (OGB-1 AM; Life Technologies, Grand Island, NY, USA) was dissolved in DMSO with 20% pluronic acid and mixed in ACSF containing 40 mM Sulforhodamine101 (SR101; Sigma-Aldrich, St. Louis, MO, USA). A glass pipette (tip diameter, 3–5 μm) was filled with this solution and inserted into the cortex to a depth of approximately 250 μm from the brain surface. The solution was pressure-ejected from the pipette (5–10 psi for 3–5 s, 7–10 times; Picospritzer III, Parker Hannifin, NH, USA). The loading site was determined using intrinsic optical imaging before craniotomy (see “Intrinsic Optical Imaging” Section). Along dorsoventral axis in the retinotopy map, we defined an area within approximately ±5° altitude as middle V1, and area of >5° and <−5° altitudes as posterior and anterior V1, respectively. We injected OGB-1 AM to the anterior, middle and/or posterior V1. OGB-1 AM was fully loaded in the cortical somata approximately for 30 min. After loading was confirmed, the craniotomy was sealed with a glass coverslip, and the imaging experiment started. Data were not recorded from all the three points in the same mouse due to technical problems.

Changes in Ca^2+^ fluorescence in cortical cells were monitored using a two-photon microscope (LSM7MP, Zeiss, Oberkochen, Germany) equipped with a mode-locked Ti:sapphire laser (MaiTai Deep See, Spectra Physics, Santa Clara, CA, USA) and a 25× objective (NA: 1.05, XL Plan N, Olympus, Tokyo, Japan). The average laser power delivered to the brain was <35 mW, depending on depth of the focus. OGB-1 AM and SR101 were excited at 920 nm. The emission filters were 470–550 nm for OGB-1 AM and 600–650 nm for SR101. The microscope objective and the photomultipliers were shielded from stray light. Images were acquired using Zeiss Zen software. A square region of cortex (190–260 μm on each side) was imaged at 256 × 256 pixels, with a frame rate of 5 Hz. Images were obtained from 150–330 μm depths from the brain surface (layers 2/3), and the imaged planes were separated by at least 25 μm interval.

### Intrinsic Optical Imaging

After mice were anesthetized with isoflurane (3.0% for induction, 1.5–2.0% during surgery, and 0.5%–1.0% during imaging), a metal plate was attached to the skull as described above (see “Two-Photon Imaging” Section). Silicon oil was dropped on the skull to increase light transmission. Red light (620 nm) was used as excitation light. Intrinsic signals were imaged using a microscope (Me600, Nikon) equipped with a 4× objective (Plan Apo, Nikon) and recorded at 5 Hz with a CCD camera (Adimec-1000, Adimec, Eindhoven, Netherland) operated by Imager 3001 system (Optical Imaging Ltd., Rehovot, Israel).

### Wide-Field Ca^2+^ Imaging

For *in vivo* wide-field Ca^2+^ imaging, we generated transgenic mice with cortical excitatory cells that expressed the genetically encoded Ca^2+^ sensor GCaMP3. In brief, Emx1-IRES-Cre mice (Gorski et al., 2002, JAX stock # 005628) were crossed with Ai38 mice (Zariwala et al., 2012, JAX stock # 014538) to produce F1 hybrids. This transgenic mouse was used to observe activity of entire V1 because of its uniform expression of GCaMP3 over the cortex, and because it was difficult to entirely cover the V1 by tilling injections of OGB-1 AM. On the other hand, this transgenic line is not suitable for cellular level analysis. GCaMP3 fluorescent signal is relatively weak and visual responses can be detected only in small population of neurons under our anesthetized condition (Murakami et al., 2015), even though the response is enough for areal scale wide-filed imaging. Therefore we used the transgenic mouse for wide-field imaging and OGB-1 AM loading method for two-photon cellular-scale imaging. After the cranial window was made, the transgenic mouse was put under a macro zoom fluorescence microscope (MVX-10, Olympus, Tokyo, Japan) equipped with a 2× objective (MVX Plan Apochromat Lens, NA 0.25, Olympus). GCaMP3 was excited using a 100 W mercury lamp through a GFP mirror unit (U-MGFPHQ/XL, Olympus; excitation peak, 488 nm; emission peak, 507 nm). The intensity of the excitation light was adjusted using ND filter. Signals were acquired using a sCMOS camera (Zyla-4.2P-CL10, Andor Technology, UK) controlled by NIS-elements BR (Nikon). A square region of the cortex (4.2 mm on each side) was imaged at 512 × 512 pixels with a frame rate of 5 Hz.

### Color Stimulus Calibration

The power spectra of opsin-isolating stimulations were measured using a spectroradiometer (Ocean Photonics, Tokyo, Japan), and the stimulation amplitudes were adjusted between S- and M-opsin isolating stimulations according to the photoisomerization rate. We estimated the photoisomerization rate I (R*/photoreceptor/s) using the following equation (Lyubarsky et al., 1999; Naarendorp et al., 2010; Tan et al., 2015):

(1) $$\text{I}\;=\;\frac{A_{\text{pupil}}}{A_{\text{retina}}}\int_{\text{λ1}}^{\text{λ2}}{\text{F}}_{\text{cornea}}(\text{λ})\tau_{\text{media}}(\text{λ}){\text{a}}_{\text{c},\text{end-on}}(\text{λ})\text{dλ}$$

where A_pupil_ is the pupil area under the experimental condition of color stimulation without imaging (0.28 mm^2^: recorded from three mice using an infrared camera); A_retina_ is the area of the retina occupied by our color stimulation (2.8 mm^2^: calculation was following Naarendorp et al., 2010); *λ*1 and *λ*2 are the lower and upper bounds of the measuring range of the spectroradiometer (360 nm and 800 nm, respectively); F_cornea_(*λ*) (photons/μm^2^/s) is the measured flux density at wavelength *λ* of the color stimulation; τ_media_(*λ*) is the transmission of the mouse lens at wavelength *λ* (Jacobs and Williams, 2007); and a_c, end-on_(*λ*) is the end-on collecting area, the light-capture area provided by total pigment. a_c, end-on_ was formulated by the following equation (Baylor et al., 1984; Lyubarsky et al., 1999):

(2) $${\text{a}}_{\text{c},\text{end-on}}(\text{λ})\;=\;\text{f}\frac{\pi {\text{d}}^{2}}{\text{4}}(1-10^{-\Delta \text{D}(\text{λ})\text{L}})\text{γ}$$

where f is a factor of the funneling effect of the inner segment (rod, 1.24; cone, 7); d is the diameter of outer segment of photoreceptor (rod, 1.8; cone, 1.5 μm); ΔD(*λ*) is the axial density of a specific pigment at wavelength *λ*; L is the axial length of the outer segment (rod, 25; cone, 13 μm); and γ is the quantum efficiency of photoisomerization (0.67 for rod and cone). We assumed that ΔD(*λ*) is proportional to the sensitivity spectrum of a specific pigment and is represented as a product of axial density at peak wavelength (0.015 optical density/μm for rod and cone) and the sensitivity spectrum obtained from previous studies (Lyubarsky et al., 1999; Govardovskii et al., 2000; Nikonov et al., 2006).

### Visual Stimulation

**Figure 1:**
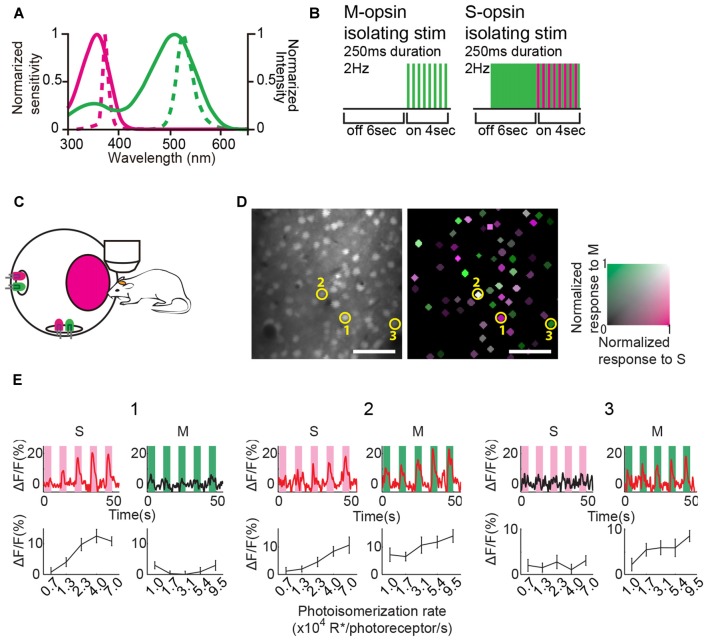
**Color stimulation design and responses of representative V1 neurons to color stimulation. (A)** Normalized spectral sensitivity of mouse S and M opsins (solid magenta and green lines, respectively), and power spectra of light emission diodes (LEDs) (broken magenta and green lines represent UV and green LEDs, respectively). **(B)** Schematics of color stimulation protocols. Color stimulation was presented for 4 s after a 6 s blank period. LEDs flashed for 250 ms at 2 Hz during the stimulation. In the S-opsin isolating stimulation trial, the green light was presented before UV stimulation, and then the intensity of the green light was weakened when the UV light flashed so that M-opsin activity was maintained before and during stimulation. **(C)** Schematic of color stimulation apparatus. An integrating sphere containing LEDs was mounted 3 cm away from the mouse eye to provide uniform color stimulation. LEDs were covered by reflecting plate (not drawn) so that LED’s light did not directly reach the mouse eye. **(D)** (Left) An example of two-photon fluorescence image. Neurons were labeled with Oregon Green 488 BAPTA-1 AM (OGB-1 AM). (Right) Neuronal responses to color stimulations represented by the green–magenta color code (green, M response; magenta, S response). Colored neurons significantly responded to at least one of the two color stimulations. The color code indicates the response magnitude normalized to the maximal response to S- or M-opsin isolating stimulation within the imaging field. Scale bars = 50 μm. **(E)** Ca^2+^-signal time courses (top row) and mean ΔF/F (bottom row) of three representative neurons indicated by the circle and number in **(D)**. Time courses were generated by averaging the data of 15 trials. Magenta and green shades illustrate stimulation periods. Stimulation intensity was varied in five steps. The red lines indicating the time courses represent a statistically significant response. Error bars indicate standard error of mean (SEM).

UV and green light emission diodes (LEDs; NSPU510CS and NSPG500DS, Nichia, Tokushima, Japan) were used as light sources for color stimulations. LEDs were attached inside an integrating sphere with a 2.5″ circular window to produce a spatially uniform and spectrally well-mixed light source (Labsphere, North Sutton, NH, USA). The circular window of the integrating sphere was located 3 cm away from the mouse eye and covered 65° of the visual field. To suppress rod activity, eyes were light-adapted with white LED attached to the integrating sphere (3.1 × 10^2^ R*/rod/s). This white light did not significantly affect cone activity because of its low luminance (Wang et al., 2011). Each experimental trial started with a blank period (6 s), and a color stimulation was subsequently flashed at 2 Hz (pulse duration, 250 ms; stimulation period, 4 s). The stimulation amplitude was changed in five steps for each color (i.e., stimulus type, 2 colors × 5 amplitudes). The I(R*/photoreceptor/s) of S-opsin isolating stimulations were varied in the range of 7.3 × 10^3^–7.0 × 10^4^ for the S opsin, 2.9 ± 0.17 × 10^4^ (mean ± SD) for the M opsin, and 1.1 × 10^4^–1.7 × 10^4^ for the rod; M-opsin isolating stimulations, 340 ± 140 for the S opsin, 1.0 × 10^4^–9.5 × 10^4^ for the M opsin, and 3.5 × 10^3^–3.4 × 10^4^ for the rod. Each stimulus was presented once every 10 trials in pseudorandom order. This cycle was repeated 15 times at each recording site. According to the mouse opsin sensitivity spectra (Figure 1A; Jacobs et al., 1991; Lyubarsky et al., 1999; Nikonov et al., 2006), the UV stimulation activated both S and M opsins (see above). To minimize this effect on the UV-evoked response, we used a color-substitution protocol (Figure 1B; Estévez and Spekreijse, 1982). In this protocol, the green light was presented before UV stimulation, and the intensity of the green light was decreased during the UV flash, depending on its amplitude, so that activation of M opsin was almost constant before (3.0 × 10^4^ R*/cone/s) and after UV stimulation (2.9 ± 0.17 × 10^4^ R*/cone/s). Only maximum amplitudes of S- and M-opsin isolating stimulations were used in the wide-field Ca^2+^ imaging.

For retinotopy mapping, a vertically-drifting horizontal white bar (5° wide, 20 s/cycle, total 16 cycles) was presented on a 37″ LCD monitor using a custom-written program running on PsychoPy (Peirce, 2007). The monitor was placed 20 cm from the right eye at 45° to the long axis of the mouse.

### Data Analysis

Data were analyzed using custom programs written in Matlab (Mathworks, Natick, MA, USA). In the analysis of two-photon Ca^2+^-imaging data, imaged frames were realigned by maximizing the correlation between frames. The contours of cell bodies were automatically identified through a series of morphological filters according to intensity, size and shape. Cell labeling was visually inspected and manually corrected. The time course of individual cells was extracted by averaging pixel intensities within cell contours. The slow drift of the baseline signal was removed using a low-cut filter (Gaussian; cut-off, 1.6 min), and high frequency noise was removed using a high-cut filter (1st-order Butterworth; cut-off, 1.6 s). To minimize neuropil signal contamination, the time course of the background signal acquired from the surrounding region of the cell contour was subtracted from the cell’s time course after multiplying by a scaling factor (Kerlin et al., 2010). The scaling factor was calculated as the ratio of mean fluorescent signal within a blood vessel to that of the surrounding background signal, averaged among several blood vessels in a FOV. The signal time course was transformed to a ratio using mean signals during inter-stimulus intervals (F). Mean ratio change (ΔF/F) during stimulation period was used in further analyses.

Responsive neurons were defined as neurons that showed statistically significant response (*P* < 0.05 by one-way ANOVA across 6 mean fluorescence values (one blank and five-intensity color stimuli in each stimulation type)) and whose maximal ΔF/F across five-intensity stimuli was more than 3%. This was computed in each stimulation separately (Tan et al., 2015), and neurons that met above criteria at least for one stimulation were defined as responsive. The color selective index (CSI) was calculated as the color preference as follows: CSI = (R_M_ − R_S_)/(R_M_ + R_S_), where R_M_ and R_S_ are the maximum responses to M- and S-opsin isolating stimulation across five intensities, respectively. A color-coded cell map was generated using the R_M_ and R_S_ of responsive cells, which was normalized to the higher value of the R_M_ and R_S_. Data were classified according to their retinotopic position of recording sites into the anterior (altitude: <−5°), middle (within approximately ±5°), and posterior V1 (>+5°), and pooled across animals.

For the stimulation of intrinsic imaging (i.e., periodically moving bar), signal intensity of responding pixel also changed periodically dependent on the bar position. In the signal time course, phase and power corresponding to the stimulus cycle represent response timing and response magnitude, respectively. The phase and power for the frequency of stimulation cycle were computed by discrete Fourier transform in each pixel, and used for drawing hue and brightness on a retinotopy map, respectively.

For the analysis of wide-field Ca^2+^ imaging data, images were averaged across trials and converted to a fluorescence ratio-change (ΔF/F) stack, using the baseline (F), mean of 1-s intervals before stimulus onset. The activation map was calculated by averaging the ΔF/F stack during the first 3 s of the stimulus periods. For each mouse, we manually set the contour of V1 region using the retinotopic map as a reference. V1 regions of activation maps were extracted, aligned according to their centroids, and averaged across animals. The activation map of M-isolating stimulus was subtracted from that of S-isolating stimulus to obtain the subtraction map. Minimum and maximum points on the averaged subtraction map were detected, and square regions (10 pixels on each side) around these points were defined as the region of interest (ROI). Time course and response amplitude were computed for each mouse by computing the average pixel value in ROIs.

### Statistical Analyses

All data are presented as the mean ± standard error of mean (SEM), unless stated otherwise. We set significance level at 0.05. When more than two groups were compared, ANOVA was used. For two-way ANOVA (2 stimulations (M vs. S) × 2 brain positions (anterior vs. posterior V1)), a significant interaction was followed by the simple effect analysis, in which the effect of one factor was tested under a specific level of another factor (e.g., M vs. S at anterior V1). After significant effect of ANOVA, Tukey’s HSD method was used for *post hoc* multiple comparisons when more than two groups were compared. For the comparisons of CSI distributions among the three V1 regions, a Kolmogorov–Smirnov test was repeated, and the *p* value was corrected by the number of repetitions (i.e., Bonferroni correction).

## Results

### Color-Responsive Neurons in V1

We used UV and green LEDs as light sources to stimulate S and M opsins (Figure 1A). LEDs were attached inside an integrating sphere with a circular window to produce a spatially uniform and spectrally well-mixed light source (Figure 1C, for details see “Materials and Methods” Section). For M opsin activation, only the green light was flashed during the stimulation period (Figure 1B, left), because green light activates only M opsins (Figure 1A). This stimulation was termed M-opsin isolating stimulation. According to the mouse opsin-sensitivity spectrum, UV light activates not only S opsins but also M opsins (Figure 1A; Sun et al., 1997; Yokoyama et al., 1998; Lyubarsky et al., 1999; Govardovskii et al., 2000; Nikonov et al., 2006). However, simple UV light sources were used in previous studies of color processing in the mouse V1 (Ekesten and Gouras, 2008; Tan et al., 2015). To dissociate S opsin activity from UV-evoked component, we used a color-substitution method (Figure 1B, right; Estévez and Spekreijse, 1982). For S opsin activation, green light was presented during a blank period before UV stimulation, and then green light was weakened while UV light was turned on (Figure 1B, right), so that M opsin activity by the UV and green light was almost constant before and during stimulation periods. Then, S opsin activity was obtained by subtracting of baseline activity from activity during stimulation period. This method allowed us to minimize the contamination of M opsin activity and isolate mainly S-opsin activity from the UV-evoked component. This stimulation was termed S-opsin isolating stimulation.

**Figure 2:**
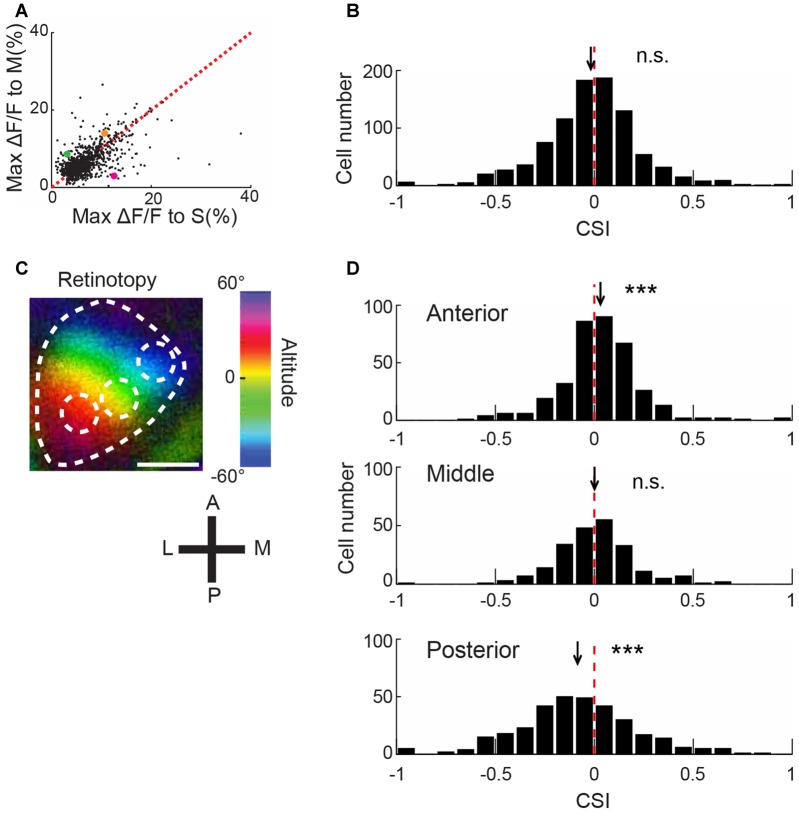
**Biased color preference of V1 neurons depending on the retinotopic position. (A)** Scatter plot of maximal ΔF/F between color stimulations. The maximum value across five intensity stimuli was plotted for each neuron (*n* = 910). Magenta, green and orange dots represent cells 1, 2 and 3 shown in Figure 1E, respectively. The broken line represents *y* = *x*. **(B)** Distribution of the color selective index (CSI). The broken line represents *x* = 0. Many neurons responded more or less to both color stimulations, and only a small number of neurons exclusively responded to one stimulation (3% for neurons with an absolute value of CSI >0.6). *n* = 910 neurons. n.s., not significantly different from 0 by signed rank test. **(C)** A representative retinotopy map. The mouse was presented with a vertically-moving horizontal bar on the monitor, and the activity of V1 was recorded using intrinsic optical imaging. Color brightness represents the response magnitude, and color hue represents the height of the bar. In this example, OGB1-AM was injected at the center of white broken circles. Scale bar = 1 mm. **(D)** CSI histogram of neurons in the anterior, middle and posterior regions of V1. Red broken lines represent 0. The positions of black arrows represent the mean CSI. CSI values were significantly different from 0 and biased to positive and negative values in the anterior and posterior regions, respectively, indicating a relatively strong response to M- and S-opsin isolating stimulations in the anterior and posterior regions (*n* = 359, 222 and 329 neurons from 7, 3 and 4 mice, respectively). n.s., not significant; ***p < 0.001 by signed rank test.

To monitor neuronal activity, we injected OGB1-AM into layers 2/3 of V1 and recorded Ca^2+^ responses to color stimulation using two-photon microscopy (Figure 1D). We found that some neurons in V1 responded to the S-and/or M-opsin isolating stimulation with diverse preferences. The neurons responsive to S- and/or M-opsin isolating stimulation were statistically defined based on the responses to five intensities of S- or M-opsin isolating stimulations (*p* < 0.05 by one-way ANOVA) and their maximum responses (maximum ΔF/F > 3%) at least for one stimulation type. Figure 1E shows example neurons that respond only to S-opsin isolating stimulations (cell 1), M-opsin isolating stimulations (cell 3), and both S- and M-opsin isolating stimulations (cell 2). Among the 2820 neurons in 20 imaging fields in anterior, middle and posterior V1 from 10 mice, we found that 30.4% ± 4.5% (mean ± SEM) neurons were responsive at least to the one stimulation. We compared responses (ΔF/F) to S- and M-opsin isolating stimulations (Figure 2A). Many neurons were distributed near the diagonal line, indicating that these neurons responded to both S- and M-opsin isolating stimulations with different sensitivities, and small populations of neurons responded exclusively to a single stimulation.

To evaluate the degree of color preference, we calculated an index for each neuron (CSI, see “Materials and Methods” Section). If the index is approximately −1 or 1, a neuron exclusively responds to S- or M-opsin isolating stimulation, respectively. For example, CSIs of cell1, 2 and 3 in Figure 1D are −0.615, 0.135 and 0.469, respectively. Distributions of CSI appeared to follow a normal distribution (Figure 2B). These results suggest that neurons in the mouse V1 have various degrees of color preference in the normal distribution manner.

### Retinotopic Position-Dependent Bias of Color Representation in V1

The distributions of S and M opsin expressions are biased along the dorsoventral axis in the mouse retina, where these opsins are dominant on the ventral and dorsal sides, respectively (Szél et al., 1992; Applebury et al., 2000). If this gradient distribution is reflected by the neuronal responses in V1, we expect that V1 neurons exhibit a biased response along the dorsoventral axis in the retina. An electrophysiological study supports this expectation (Ekesten and Gouras, 2008). In contrast, a two-photon imaging study found no bias of color representation in the mouse V1 along mediolateral axis (Tan et al., 2015), but color representation along anteroposterior axis was not examined. Comparison of retinotopy data in different articles shows that dorsoventral axis of visual field is slightly different between the articles (Kalatsky and Stryker, 2003; Marshel et al., 2011), suggesting a possibility that mediolateral axis in V1 does not precisely correspond to the dorsoventral axis in the retina and the bias of color representation may exist along a cortical axis, which corresponds to the dorsoventral axis of the retina. We identified the cortical axis in individual mice by retinotopy mapping using intrinsic optical imaging (Figure 2C), which showed that the anterior region of V1 received input from the dorsal retina (M opsin dominant), and the posterior region received input from the ventral retina (S opsin dominant).

We conducted two-photon imaging of the different regions along the anteroposterior axis of V1. After imaging, we analyzed the data acquired from the anterior, middle and posterior regions, separately. This analysis revealed that biased color representation is consistent with the opsin gradient in the retina. In the anterior V1 average of CSI was biased to 1, whereas that of the posterior V1 was biased to −1 (anterior: *p* = 2.4 × 10^−4^, middle: *p* = 0.98, and posterior: *p* = 2.4 × 10^−7^ by signed rank test; *n* = 359, 222 and 329 neurons, respectively; Figure 2D). Comparisons of CSI scores between anterior and posterior V1 showed a significant difference (*p* = 4.8 × 10^−14^ by Kolmogorov–Smirnov test with Bonferroni correction). Similar CSI bias of anterior and posterior V1 were observed in the data from the same mice (Supplementary Figure S1). These results indicate that cortical position affects color preference in V1 and suggest that the color representation along anteroposterior axis probably reflects the dorsoventral distribution of opsins in the retina.

### Wide-Field Ca^2+^ Imaging Confirms Biased Color Representation

**Figure 3:**
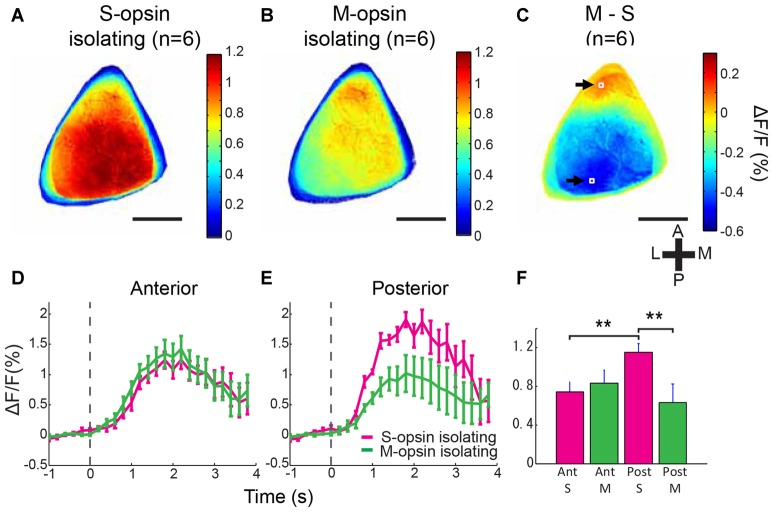
**Biased color representation of entire V1. (A,B)** Averaged activation maps to S- and M-opsin isolating stimulations in V1 (*n* = 6 mice). Outline of V1 was determined for each animal according to the retinotopy and activation maps of grating patch, flashed bar and color stimulations. Extracted V1 images were aligned by the centroid of each V1 regions and averaged among animals. S-opsin isolating stimulation evoked strong activity in the posterior region **(A)**, whereas M-opsin isolating stimulation activated mainly anterior part of V1 **(B)**. **(C)** Averaged subtraction map (Panel **B** minus Panel **A**) demonstrates the S-opsin and M-opsin response-dominant region (positive and negative values represented by the color code indicate M-opsin- and S-opsin-response dominant areas, respectively). Scale bars = 1 mm. **(D,E)** Averaged response time courses from anterior and posterior regions shown in **(C)** by the white boxes indicated by black arrows. These two were minimal (S-opsin dominant) and maximal (M-opsin dominant) points, which were detected in the averaged subtraction map **(C)**. Magenta and green lines represent responses to S- and M-opsin isolating stimulations, respectively. Stimulation was presented from 0 s to 4 s. Broken line represents stimulation onset. **(F)** Comparison of mean ΔF/F. Response to S-opsin isolating stimulation in posterior V1 was significantly higher than response to S-opsin isolating stimulation in anterior and response to M-opsin isolating stimulation in posterior. **p < 0.01 by two-way repeated measured ANOVA followed by simple effect analysis test (*n* = 6 mice). Scale bars in **(A–C)** = 1 mm.

We have shown a single cell-level bias of color responsiveness recorded by the two-photon Ca^2+^ imaging, whose recording area is limited to several hundreds of microns. To study functional organization of color bias across V1, we performed wide-field Ca^2+^ imaging (Murakami et al., 2015). This technique enables us to monitor the color response over V1 (Figure 3). We recorded from a transgenic mouse that expressed the Ca^2+^ sensor GCaMP3 (Tian et al., 2009) mainly in cortical excitatory neurons (see “Materials and Methods” Section).

In the averaged activation maps across six mice, S-opsin isolating stimulation evoked a stronger response in the middle and posterior V1, whereas M-opsin isolating stimulation activated the anteromedial V1 with a stronger response in the most anterior region (Figures 3A,B). In the averaged subtraction map, the S-opsin-response-dominant region covered most of the posterior two-thirds of V1 responsible for the upper visual field, whereas the M-opsin-response-dominant region was localized to the most anterior V1 region responsible for the lower visual field (Figure 3C; Wang and Burkhalter, 2007; Garrett et al., 2014). Similar tendency was observed in the individual mouse (Supplementary Figures S1B–D). The maximum and minimum points of the subtraction map were located in the anterior and posterior V1 regions, respectively (white boxes and arrows in Figure 3C), and ROIs for statistical analysis were set around the two points. The time courses and mean responses were computed from the two ROIs (Figures 3D–F). The S-opsin response in the posterior region was significantly higher than the M-opsin response in the posterior region and the S-opsin response in the anterior region (Figure 3F; *p* = 0.001 for S-opsin vs. M-opsin isolating stimulation in the posterior V1 and *p* = 0.0066 for anterior vs. posterior regions in S-opsin isolating stimulation; significant interaction by two-way (2 stimulations × 2 positions) repeated measures ANOVA followed by simple main effect analysis). These results are consistent with those obtained using two-photon Ca^2+^ imaging and confirm the biased color representation in the anterior and posterior V1 regions that reflect the gradient of opsin expression in the retina.

### Color-Responsive Neurons Intermingle within Several Hundred Microns

**Figure 4:**
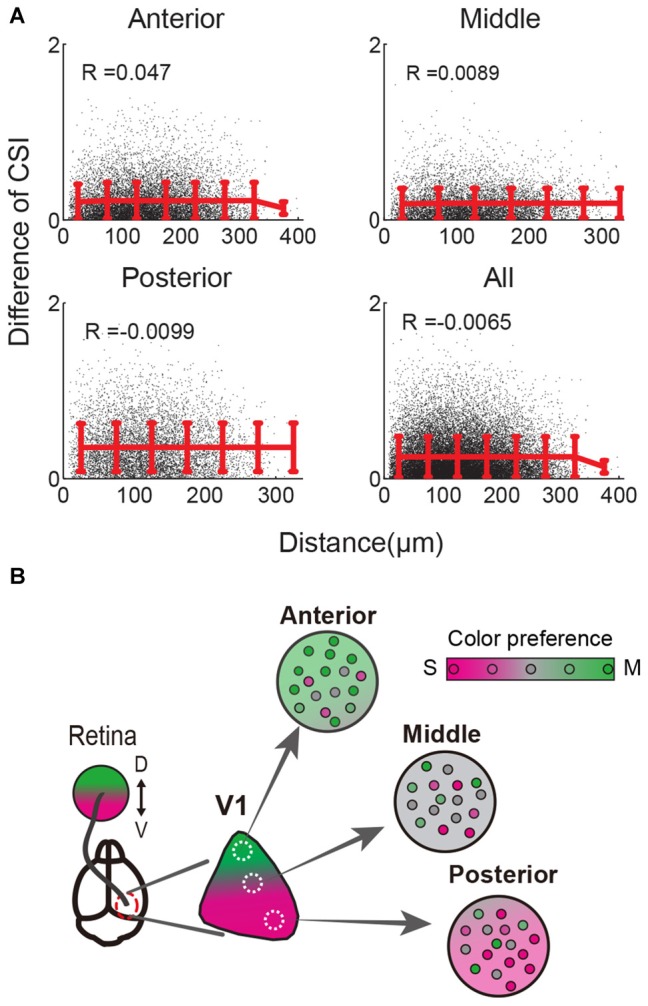
**Spatial distribution of color responsive neurons in the local area. (A)** Distances and CSI differences of all color-responsive neuron pairs were calculated and plotted. A single black dot indicates the data for each pair. Red lines and error bars indicate the mean values and SD of black dots binned every 50 μm. Pearson’s correlation coefficients (R) are shown. The mean CSIs are almost constant independent of the distance between pairs in all three V1 regions and all data (bottom right). These results indicate that color preferences are intermingled at the single cell level within several hundred microns. **(B)** Scheme of local and retinotopically dependent color representation. Population average of color preference changed depending on the retinotopic position (three circles: Anterior, Middle, Posterior, indicate local area of V1, and their background colors represent local averages of color preferences). In contrast, color preferences of single neurons were variable and locally intermingled (represented by small circles in each local area). This across-cell variability is higher than change of the averaged color preference depending on the retinotopic position in the range of several hundred microns (Background color change very slightly in each local area). Thus CSI differences between cells did not depend on the distance within several hundred microns in **(A)**.

A recent study reported that color-responsive neurons were intermingled within a single imaging field encompassing several hundred microns in the center of mouse V1 (Tan et al., 2015). However, it remains unknown whether intermingled distribution of color-responsive neurons is also found in the anterior or posterior part of V1 where the color response is biased. Therefore, we addressed this issue with our data obtained from the anterior and posterior V1. In an example of a color-response map recorded from posterior V1, S- and M-opsin responsive neurons were spatially intermingled (Figure 1D). For example, an S-opsin responsive neuron (indicated by “1”) was surrounded by both S- and M-opsin responsive neurons, whereas an S- and M-opsin responsive neuron (indicated by “2”) was surrounded by both S-opsin responsive neurons and M-opsin responsive neurons (Figure 1D). This suggests that neurons with different color preferences are spatially intermingled even outside the center of V1. To quantify the spatial distribution of color-responsive neurons, we calculated the differences of the CSI values for all pairs and examined its relationship with the distance between neurons (Figure 4A). If neurons with similar color preference form clusters, CSI differences between neighboring neurons will be small. In contrast, if they do not form a cluster, CSI differences will uniformly distribute independent of the distance. Our results showed that CSI differences were uniformly distributed in the anterior, middle, and posterior regions of V1 as well in the total population (Figure 4A). These results indicate that color-responsive neurons with various color preference are intermingled independent of location in V1.

## Discussion

We demonstrated that neurons in layers 2/3 of the mouse V1 showed a biased color representation along the anteroposterior axis of V1, which is consistent with the pattern of opsin expression in the retina. This biased color representation was confirmed by functional wide-field imaging over V1. In contrast, S- and M-opsin isolating stimulation responsive neurons are intermingled in the range of several hundred microns independently of V1 region. These results indicate that average of color preferences across local population depends on retinotopic position, but a minority which prefers the other color exists and is locally intermingled with the majority. Our findings show the functional organization of color representation in mouse V1.

### Technical Differences from Previous Studies

Two previous studies described the color representation in mouse V1 (Ekesten and Gouras, 2008; Tan et al., 2015). Compared to these studies, our study used different technical strategies to determine local and areal color representations. The first difference was the stimulus protocol. Stimulation with UV light activates both S and M opsins because M opsin has weak side-band sensitivity to UV light, in addition to its peak wavelength of approximately 510 nm (Sun et al., 1997; Yokoyama et al., 1998; Lyubarsky et al., 1999; Govardovskii et al., 2000; Nikonov et al., 2006). To minimize this contamination, we used a color-substitution protocol to maintain constant M opsin activation during a S-opsin isolating stimulation trial (Estévez and Spekreijse, 1982). This was not used in the previous mouse studies (Ekesten and Gouras, 2008; Tan et al., 2015). The second difference was the functional identification of recording sites by retinotopic mapping. In contrast to the previous studies where recording sites were determined by stereotaxic coordinates, we used intrinsic optical imaging to functionally identify the dorsoventral axis of retina on the cortex. The identification of the retinotopic position of the recording sites minimized the effect of variability of V1 positions across mice. This helped us to find the biased color representation along anteroposterior axis in V1 at cellular level. Finally, to determine areal representation of the color bias, we used wide-field Ca^2+^ imaging, which is more suitable for examining areal representation. Ekesten and Gouras (2008) used single unit electrophysiology with which it is difficult to completely cover the entire V1, whereas Tan et al. (2015) recorded from small parts of V1 (see below). Thus, color representation covering whole V1 has not been reported, and our result of the wide-field imaging (Figure 3) is the first report of a color preference map which completely covers entire mouse V1.

### Biased Color Representation in the Mouse V1

In the mouse retina, S and M opsins exhibit a gradient expression pattern along the dorsoventral axis in the retina (Applebury et al., 2000). Retinal ganglion cells exhibit biased color responses depending on their location in the retina (Ekesten et al., 2000; Ekesten and Gouras, 2005; Wang et al., 2011). However, the results of previous studies of the color representation in the mouse V1 seem inconsistent (Ekesten and Gouras, 2008; Tan et al., 2015). Ekesten and Gouras (2008) reported biased distribution of UV- and green-responsive neurons consistent with the distribution of retinal opsin, whereas Tan et al. (2015) observed no bias in color representation along mediolateral axis. The discrepancy between these results is probably because of the range of recording sites along anteroposterior axis. Our wide-field imaging revealed that strongly biased color representation was restricted to the relatively small area of the anterior and posterior regions of V1, which may reside outside the recording site of Tan et al. (2015), according to their stereotaxic coordinates (~3 mm lateral, 0.5 mm anterior to lambda and ~2 mm lateral, 0.5 mm anterior to lambda). We functionally identified the dorsoventral axis of retinotopy and observed biased representation even using two-photon imaging. Thus, a precise retinotopic mapping method was crucial for identifying biased color representation in V1.

The stimulus pattern might affect the result. Our study and that of Ekesten and Gouras (2008) used flashing stimuli without a spatial structure. In contrast, Tan et al. (2015) used drifting gratings of UV and visible light. Therefore, our data together with those of Ekesten and Gouras (2008) indicate that the mouse V1 shows biased responsiveness at least to spatially uniform flash stimuli.

In this study, the bias of CSI in the anterior V1 was weaker than that in the posterior V1 although the bias was statistically significant (Figure 2D). Wide-field imaging also revealed that the bias in the anterior V1 was weak and restricted in the small anterior region (Figure 3C and Supplementary Figure S1). The weak bias in the anterior V1 could be due to the pattern of opsin expression. The expression ratio of S and M opsins in a single cone is relatively equivalent on the dorsal side of retina, and the M-opsin dominant cone is found only in the upper edge of dorsal retina, whereas S opsin is dominant in the broad area of ventral retina (Applebury et al., 2000). Consistent results were obtained by electrophysiological recording from retinal ganglion cells. Dorsal retinal ganglion cells showed relatively similar sensitivity to UV and green light, whereas ventral cells were very sensitive to UV light (Wang et al., 2011). These suggest that the M-opsin-dominant bias is weak and spatially limited, when compared with the S-opsin-dominant bias in the retina, and that this color representation in the retina is well preserved in the V1. Furthermore, according to the retinotopy map, the cortical area corresponding to the M-opsin dominant retinal area is very small, making recording with 2-photon microscopy potentially difficult (see Wang and Burkhalter, 2007; Garrett et al., 2014 for retinotopy of the mouse). This may result in the smaller bias in the anterior V1 (Figures 2D, 3F).

### Color Response of Single V1 Cells

Consistent with the previous studies (Ekesten and Gouras, 2008; Tan et al., 2015), many V1 neurons responded to S- and M-opsin isolating stimulations with variable preferences, and small population of cells was exclusively selective to one color, independent of the location in V1. We observed that the strength of color preference largely follows a normal distribution, indicating that many neurons responded to both stimuli with various color preference and the average of the color preference changed along anteroposterior axis in V1.

As qualitatively described in the previous study (Tan et al., 2015), our quantitative analysis also suggests that, at a cellular level, color preferences are spatially intermingled in the range of several hundred microns in mouse V1. This “salt and pepper-like” pattern is similar to other visual features of rodent V1, i.e., orientation and direction selectivity (Ohki et al., 2005), receptive field structures (Smith and Häusser, 2010; Bonin et al., 2011), binocular disparity (Scholl et al., 2013), and eye-specific responses (Mrsic-Flogel et al., 2007; Scholl et al., 2013). This result may seem to be inconsistent with the results of wide field imaging. Because population average of color preference changed depending on the retinotopic position, it would be possible that neighboring cells tend to share similar color preference. However, CSIs were variable across cells and locally intermingled. Compared to this variability, CSI change along anteroposterior axis of retinotopy was small in the range of several hundred microns (Figure 4B). Thus CSI differences between cells did not depend on the distance within several hundred microns.

### Comparison with Trichromatic Primates

Trichromatic primates have many different visual properties compared to rodents (Huberman and Niell, 2011). One big difference is an existence of functional domains for color processing, called blobs. These blobs contain color responsive neurons such as double opponent cells (Livingstone and Hubel, 1984; Lu and Roe, 2008) and it is generally believed that double opponent cells are important for color contrast and constancy (Conway, 2009). In contrast mice do not have such functional domains and color responsive neurons are distributed throughout V1. On the other hand, color opponency in mouse V1 has not yet to be fully examined and needs been resolved for further understanding of color processing in mice.

In summary, our results demonstrate that color preferences are represented in a “salt-and-pepper” pattern with a retinotopic gradient of color bias in mouse V1.

## Author Contributions

SA, TY and KO designed the research. SA performed two photon imaging and optical intrinsic imaging experiments. TY performed wide-field Ca^2+^ imaging experiment. SA, TY and TH analyzed the data. SA, TY and KO wrote the manuscript. All authors discussed the results and commented on the manuscript.

## Conflict of Interest Statement

The authors declare that the research was conducted in the absence of any commercial or financial relationships that could be construed as a potential conflict of interest.
