# Supplementary material for: Color Representation Is Retinotopically Biased but Locally Intermingled in Mouse V1
Source: Front Neural Circuits. 2017 Mar 29;11:22. doi: 10.3389/fncir.2017.00022 (PMC5370321; doi:10.3389/fncir.2017.00022)
Supplement: Supplementary file 1 [file Image_1.pdf]

Supplementary Material

Color representation is retinotopically biased but locally intermingled in mouse V1

Shuhei Aihara[1, †], Takashi Yoshida[1, 2], Takayuki Hashimoto[1] and Kenichi Ohki[1,2,3,*]

[1]Department of Molecular Physiology, Graduate School of Medical Sciences, Kyushu University, Fukuoka, Japan

[2]Department of Physiology, The University of Tokyo School of Medicine, Tokyo, Japan.

[3]CREST, Japan Science and Technology Agency, Tokyo, Japan

***Correspondence:** Kenichi Ohki: kohki@m.u-tokyo.ac.jp, Takashi Yoshida: takashiy@m.u-tokyo.ac.jp

[†]Present address: Laboratory for Sensory Circuit Formation, RIKEN, Center for Developmental Biology, Kobe, Japan

1 Supplementary Figures

Here we show one supplementary figure related to Figure 2 and 3.

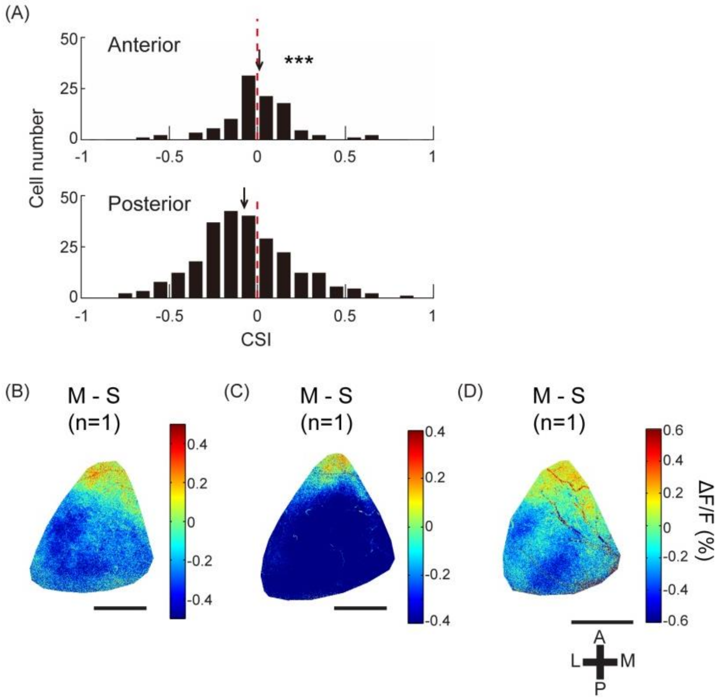

Supplementary Figure 1. Results of individual mice: CSI histograms and wide-filed subtraction maps. (A) CSI histograms from two mice in which recording were performed from both anterior and posterior V1 in the same mouse. These two histograms showed a similar tendency to that of population data. ***: $P < 0.001$ by Kolmogorov Smirnov test. (B-D) Three examples of subtraction map from single mice. Anterior V1 showed positive value (M-opsin response dominant), whereas posterior V1 showed negative value (S-opsin response dominant), indicating that graded color representation was consistent across mice.
